# Predicting AH1N1 2009 influenza epidemic in Southeast Europe

**DOI:** 10.3325/cmj.2011.52.115

**Published:** 2011-04

**Authors:** Mladen Smoljanović, Ankica Smoljanović, Marijana Mlikotić

**Affiliations:** 1 University of Split School of Medicine, Split, Croatia; 2 Teaching Institute of Public Health of the Split-Dalmatian County, Split, Croatia

## Abstract

**Aim:**

To use the data on the AH1N1 2009 influenza epidemic in the Southern hemisphere countries to predict the course and size of the upcoming influenza epidemic in South-Eastern Europe (SEE) countries and other regions of the World with temperate climate.

**Method:**

We used a comparative epidemiological method to evaluate accessible electronic data on laboratory-confirmed deaths from AH1N1 2009 influenza in the seasons 2009/2010 and 2010/2011. The studied SEE countries were Albania, Bosnia and Herzegovina, Bulgaria, Croatia, Greece, Hungary, Kosovo, Macedonia, Montenegro, Romania, Serbia, and Slovenia, while Southern hemisphere countries were Argentina, Australia, Chile, New Zealand, Paraguay, Uruguay, and South Africa.

**Results:**

In influenza season 2009/2010, Southern hemisphere countries with temperate climate reported 1187 laboratory-confirmed influenza AH1N1 2009 deaths (mortality rate 0.84/100 000; 95% confidence interval [CI], 0.50-1.24). SEE countries with similar climatic conditions reported 659 deaths and similar mortality rates (0.86/100 000, 95% CI, 0.83-1.10). In the whole Europe without the Commonwealth of Independent States countries (CIS, former Soviet Union), there were 3213 deaths (0.60/100 000; 95% CI, 0.65-0.93). In 2010/2011, Southern hemisphere countries reported 94 laboratory-confirmed deaths (mortality rate 0.07/100 000; 95% CI, 0.02-0.28) or only 7.9% of the previous season. SEE countries by the end of the 11th epidemiological week of 2010/2011 season reported 489 laboratory-confirmed deaths, with a mortality rate of 0.64/100 000 (95% CI, 0.26-0.96) or 74.2% of the previous season, which was significantly higher than in the Southern hemisphere countries (χ^2^_1_ = 609.1, *P* < 0.001). In Europe without CIS countries, there were 1836 deaths, with a mortality rate of 0.34/100 000 (χ^2^ = 153.3, *P* < 0.001 vs SEE countries).

**Conclusion:**

In the 2009/2010 season, SEE countries and Southern hemisphere countries had similar influenza AH1N1 2009 mortality rates. In the 2010/2011 season, the forecast of 10% increase in total mortality in SEE countries and Europe compared with the 2009/2010 season was significantly exceeded, while the expected impact of type-specific vaccines against influenza AH1N1 2009 was not achieved. Consumption of epidemic potential has greater importance for the prognosis of the course and size of influenza epidemic than the degree of vaccine immunity.

In order to plan anti-epidemic measures for infectious diseases, including influenza, it is important to predict the course of each outbreak. This is mostly done by using historical data of previous outbreaks (1). Modern means of communication, which allow simultaneous monitoring of influenza in different parts of the world, are used to collect data from the countries where an influenza epidemic has just finished to predict its course in the countries where it is yet to begin. With the outbreak of influenza AH1N1 2009, the World Health Organization (WHO) introduced laboratory monitoring of patients and deaths by detecting influenza-specific RNA by real-time reverse transcriptase-polymerase chain reaction, available in all developed countries and many developing countries (2,3). The world now has much more reliable and comparable data based on a correct diagnosis of influenza virus types than the former practice of registration of patients with influenza-like illness and the number of deaths from influenza-like illness (2-6). Laboratory confirmation of deaths facilitates the comparison of epidemic course between countries and provides more accurate forecasts. We compared the available data from the 2010/2011 influenza season with the predictions for the same season, and we specifically compared SEE countries, which used different approaches to monitor the epidemic and perform the vaccination.

## Method

We used electronically available mortality data on influenza AH1N1 2009, collected and processed by governmental institutions, international organizations, and other relevant sources that regularly register morbidity and mortality (7-67). Mortality rate indicators (laboratory-confirmed deaths from AH1N1 2009) were calculated using the UN population estimate for each country (68). SEE countries include Bosnia and Herzegovina, Bulgaria, Croatia, Greece, Hungary, Kosovo, Macedonia, Montenegro, Romania, Serbia, and Slovenia, that altogether have 76.4 million inhabitants (7-14,68). Europe includes 40 countries, without 13 Commonwealth of Independent States (CIS) countries, and has 534.1 million inhabitants. Northern hemisphere includes 69 countries without China and the countries of Central Asia, and has 1823.4 million inhabitants. Equatorial area includes 51 countries and has 1776.8 million inhabitants. North America includes United States of America (USA) and Canada and has 340 million inhabitants. The Western hemisphere (‘The New World’) includes 35 countries of the North, Central, and South America, and has 904.0 million inhabitants. The Eastern hemisphere (‘The Old World’) includes 92 countries, with 2838.0 inhabitants. The Southern hemisphere includes Argentina, Australia, Chile, New Zealand, Paraguay, Uruguay, and South Africa and has 141.7 million inhabitants.

We compared the countries and regions of the world during the 2 most recent influenza seasons: 2009/2010 and 2010/2011. As the epidemic broke out in Mexico at the end of April 2009, the data for the 2009/10 season are from May 2009 until the end of April 2010 (69). The 2010/11 influenza season started in May 2010 and ended with the 11th epidemic week of 2011 (March 20, 2011).

Chi-square test was used to test the differences between data for the two seasons, using Statistica 7 software package (StatSoft, Tulsa, OK, USA).

## Results and discussion

In Tables 1-4, the number of laboratory-confirmed influenza deaths is summarized and compared between the countries of Europe, North America, Northern hemisphere, Western hemisphere, Eastern hemisphere, equatorial area, and the world. Out of 214 world countries, with 6724.3 million inhabitants, 127 countries (3743.4 million inhabitants) adequately monitored and regularly reported on the number of laboratory-confirmed deaths.

**Table 1:** Laboratory-confirmed deaths from influenza AH1N1 09 per month in 2009/2010 season*

		Season 2009/2010												
Country		2009								2010				Total
	Population (million)	V	VI	VII	VIII	IX	X	XI	XII	I	II	III	IV	
**Albania**	3.2								**6**	**6**	0	0	0	**12**
**Bosnia and Herzegovina**	4.0							1	**8**	**2**	**8**	0	0	**19**
**Bulgaria**	7.6					1	1	**3**	**30**	**5**	0	0	0	**40**
**Croatia**	4.5						1	**9**	**14**	**5**	0	3	0	**32**
**Greece**	11.2				1	2	0	**9**	**58**	**36**	**34**	6	3	**149**
**Hungary**	10.0			1	0	1	2	3	**45**	**55**	**17**	6	4	**134**
**Kosovo**	2.1							**2**	**12**	0	0	0	0	**14**
**Macedonia**	2.1							1	**15**	**8**	2	0	0	**26**
**Montenegro**	0.7								**5**	**3**	0	0	0	**8**
**Romania**	21.5							2	**56**	**53**	**11**	0	0	**122**
**Serbia**	7.5						1	**16**	**38**	**26**	2	0	1	**84**
**Slovenia**	2.0							**2**	**11**	**6**	0	0	0	**19**
**Total South-Eastern Europe**	**76.4**			1	1	4	5	**48**	**298**	**205**	**74**	15	8	**659**
				0.2	0.2	0.6	0.7	**7.3**	**45.2**	**31.5**	**11.2**	2.3	1.2	100%
**Europe without CIS countries**	**534.1**	0	1	44	56	58	**146**	**598**	**1179**	**637**	**217**	**228**	49	**3213**
		0	0.03	1.4	1.7	1.8	**4.5**	**18.6**	**36.7**	**19.8**	**6.7**	**7.1**	1.5	100%
**Argentina**	40.5		**26**	**433**	**7**	**73**	**58**	17	4		9			**627**
**Australia**	21.7		**7**	**68**	**80**	**24**	7	4	1					**191**
**Chile**	17		**12**	**84**	**34**	2	4	12	2	3	2			**155**
**New Zealand**	4.2			**16**	**11**								8	**35**
**Paraguay**	6.8			**22**	**19**	**11**							1	**53**
**South Africa**	48			2	**25**	**32**	**32**		2					**93**
**Uruguay**	3.5		1	**24**	**5**	**3**								**33**
**Total Southern hemisphere** (7 countries)	**141.7**	**0**	**46**	**649**	**181**	**145**	**101**	**33**	**9**	**3**	**11**	**0**	**9**	**1187**
		0	3.9	54.7	15.2	12.2	8.5	2.8	0.8	0.2	0.9	0	0.8	100%
**Equatorial area** (51 countries)**^†^**	**1776.8**	**1**	**12**	**371**	**1011**	**922**	**675**	**400**	**711**	**451**	**328**	**420**	**146**	**5448**
			0.2	6.8	18.6	16.9	12.4	7.3	13.1	8.3	6.0	7.7	2.7	100%
**Northern hemisphere** (69 countries)**^†^**	**1823.4**	**114**	**161**	**401**	**382**	**1543**	**1947**	**3031**	**3599**	**1696**	**514**	**1119**	**147**	**14** **654**
		0.8	1.1	2.5	2.6	10.5	13.3	20.7	24.6	11.6	3.5	7.6	1.0	100%
**The world** (127 countries)**^†‡^**	**3743.4**	**115**	**219**	**1421**	**1574**	**2610**	**2723**	**3464**	**4319**	**2150**	**853**	**1539**	**301**	**21** **288**
		0.5	1.0	6.7	7.4	12.3	12.8	16.3	20.3	10.1	4.0	7.2	1.4	100.0%
**Total Western hemisphere** (35 countries)**^†^**	**904.0**	**115**	**205**	**1182**	**1055**	**2134**	**2107**	**1572**	**954**	**506**	**432**	**393**	**173**	**10** **828**
		1.1	1.9	10.9	9.7	19.7	19.5	14.5	8.8	4.7	4.0	3.6	1.6	100%
**Total Eastern hemisphere** (92 countries)**^†^**	**2838.0**	**0**	**14**	**239**	**519**	**476**	**616**	**1892**	**3365**	**1644**	**421**	**1146**	**128**	**10** **460**
		0	0.1	2.3	5.0	4.6	5.9	18.1	32.2	15.7	4.0	11.0	1.2	100%
**The world** (214 countries)**^§^**	**6724.3**	**115**	**219**	**1438**	**1590**	**2640**	**2741**	**3624**	**4902**	**2267**	**853**	**1542**	**302**	**22** **233**
		0.5	1.0	6.5	7.2	11.9	12.3	16.3	22.0	10.2	3.8	6.9	1.3	100%
**Total Northern America** (2 countries)	**340.0**	**17**	**135**	**322**	**212**	**1385**	**1495**	**1031**	**489**	**174**	**113**	**89**	**29**	**5491**
		0.3	2.5	5.9	3.9	25.2	27.2	18.8	8.9	3.2	2.1	1.6	0.5	100%

*Shaded fields indicate enhanced seasonal influenza activity; CIS – Commonwealth of Independent States. Source: references 7-67.

†Countries with total mortality rates in both seasons <0.10/100 000 were excluded from calculation.

‡Reliable data on influenza-related deaths were available for 127 countries.

§Calculations for all 214 countries, regardless of the number of reported deaths.

**Table 4:** Total mortality rates of laboratory-confirmed deaths from Influenza AH1N1 09 for world’s regions in both seasons

		2009/2010 season		2010/2011 season		Both seasons		2010/2011 vs 2009/2010 season	
Country	Population (million)	No. deaths	mortality per 100 000	No. deaths	mortality per 100 000	No. deaths	mortality per 100 000	number	%
Northern America (2 countries)	340.0	5491	1.62	267	0.08^†^	5758	1.69	267/5491	4.9
South-Eastern Europe (12 countries)	76.4	659	0.86	489	0.64^†^	1148	1.50	489/659	74.2
Western hemisphere (35 countries)*	904.0	10 828	1.20	756	0.08^†^	11 584	1.28	756/10 828	7.0
Northern hemisphere (69 countries)*	1823.4	14 654	0.80	2823	0.15	17 477	0.96	2823/14 654	19.3
Europe without CIS countries (40 countries)	534.1	3213	0.60	1836	0.34	5049	0.94	1836/3213	57.1
Southern hemisphere (7 countries)*	141.7	1187	0.84	94	0.07	1281	0.91	94/1187	7.9
The world (127 countries)*^‡^	3743.4	21 289	0.57	4780	0.13^†^	26 069	0.70	4780/21 289	22.5
Eastern hemisphere (92 countries)*	2838.0	10 460	0.37	4024	0.14^†^	14 484	0.51	4024/10 460	38.4
Equatorial area (51 countries)*	1776.8	5448	0.31	1853	0.10^†^	7301	0.41	1853/5448	34.0
The world (214 countries)^§^	6724.3	22 233	0.33	4945	0.07	27 078	0.40	4945/22 233	22.2

*Countries with total mortality rates <0.10/100 000 for both seasons were excluded from calculation. Sources: references 7-67.

†*P* < 0.001 vs previous season.

‡Reliable data on influenza-related deaths were available for 127 countries.

§Calculations for all 214 countries, regardless of the number of reported deaths.

The outbreak of influenza AH1N1 2009 in Mexico in April 2009 was announced as the first catastrophic pandemic – the ‘young people killer’ influenza virus – of the third millennium. With the surrounding media frenzy, the influenza scare traveled quickly around the world (13-20), and frightened governments were forced to take action by activating the pandemic plans prepared after the pandemic avian influenza in 2005 (70-77). However, the first findings from Mexico, together with the course of the epidemic in the Southern hemisphere countries where local winter was starting, indicated that there was no exceptional, out-of-season outbreak of dangerous ‘killer virus.’ Instead, it was the usual epidemic of highly contagious influenza virus AH1N1 2009, accompanied by the usual low mortality and lethality as is the case for the ordinary seasonal influenza (15-20,48-50). Indeed, the mortality in the 2009/2010 season was much lower than had been predicted by the WHO and the USA Center for Disease Control (77-79). Based on the number of laboratory-confirmed deaths from influenza in Southern hemisphere countries, the expected mortality in Northern hemisphere countries, where winter was starting, was 0.84/100 000 population. It was estimated that the epidemic would be a single-wave, lasting 10-16 weeks, with the onset 2 months earlier than usual (80). It was expected that SEE countries would have lower mortality rates than the Southern hemisphere countries, due to the availability of the type-specific vaccine against influenza virus AH1N1 09 (80-82).

After such experiences from 2009/2010 influenza season, a similar sequence of events was expected in 2010/2011 season. By that time, the South hemisphere countries had had trivalent type-specific vaccines for AH1N1 2009 influenza virus (83). The influenza season ended with a total of 96 registered laboratory-confirmed deaths (Tables 1-4). The highest mortality rate was in New Zealand (17), while Argentina, Australia, and Chile had a very low mortality rate and South Africa and Uruguay had no laboratory-confirmed deaths (15-20). The data on vaccination coverage were not available for all countries, but were reliable for New Zealand and Argentina, where about a quarter of the total population was vaccinated: 1.1 million in New Zealand and 10 million in Argentina (83-85). Despite the similar vaccination coverage, the mortality in Argentina (0.05/100 000) was significantly lower than in New Zealand (0.48/100 000). Also, the mortality in New Zealand did not differ from that in 2009/2010, despite vaccination (0.83/100 000; χ^2^_1_ = 3.56, *P* > 0.10) (15,17).

In the 2010/2011 season, Southern hemisphere countries recorded only 7.9% (95% confidence interval [CI], 3.74-33.03) of laboratory-confirmed influenza deaths, less than in the previous season; χ^2^_1_ = 930.9, *P* < 0.001).

As the influenza season in the Southern hemisphere precedes the one in the Northern hemisphere, it was expected that Northern hemisphere countries would also have a similar lower mortality rate. By the end of the 11th epidemic week (March 20, 2011, the end of influenza season), in North American countries 267 laboratory-confirmed deaths were recorded, with a mortality rate of 0.08/100 000 or only 4.7% of the 2009/10 season. According to the Centers for Disease Control weekly reports on the total number of laboratory-confirmed deaths from different types of influenza viruses, deaths from influenza virus AH1N1 2009 made up at most a third of the cumulative number of all types of influenza viruses in the USA (48). Mexico did not report any deaths caused by influenza AH1N1 2009 virus and reported a low morbidity, just confirming the presence of influenza AH1N1 2009 virus in circulation. Canada reported 13 laboratory-confirmed deaths (48-50). The total number of laboratory-confirmed deaths in the Northern hemisphere was 2823 or only 19.3% of the previous season. For all the world countries, the laboratory-confirmed influenza mortality reached 22.2% of that in the previous season.

In Europe without the CIS, all countries recorded lower laboratory-confirmed mortality in 2010/2011 than in 2009/2010 (0.34 vs 0.60). The percentage of laboratory-confirmed deaths (n = 1836) was 57.1% of that for the 2009/10 season (n = 3213). The lower mortality rate in Europe than in Southern hemisphere countries in the season 2009/2010 (0.60 vs 0.84, χ^2^_1_ = 95.5, *P* < 0.001) indirectly points to a smaller consumption of epidemic potential in that season in Europe.

**Figure 1:**
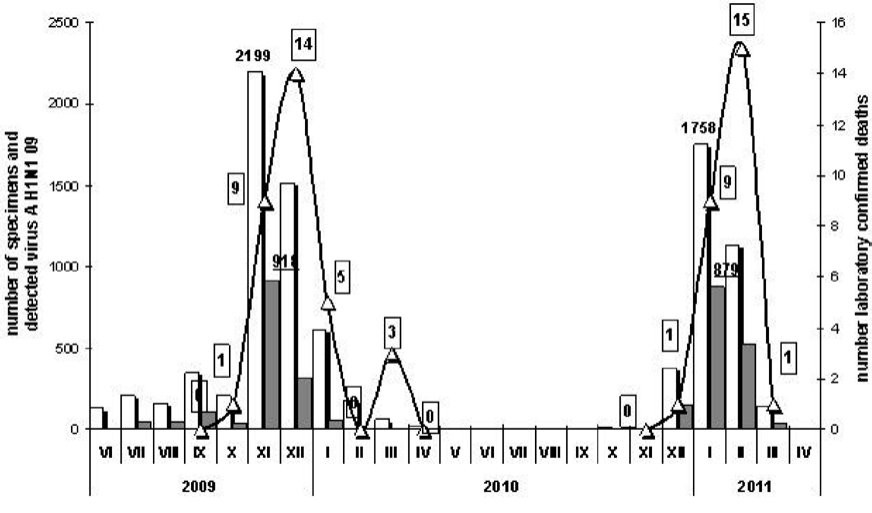
Influenza AH1N1 2009 in Croatia, number of specimens processed (open columns), number of detected influenza virus AH1N1 2009 cases (gray columns) and number of laboratory confirmed deaths (triangles and framed number). Sources: Croatian Public Health Institute (8) and WHO Flunet, *http://apps.who.int/globalatlas/dataQuery/default.asp*.

In 2009/10 influenza season, SEE countries had an average mortality rate similar to Southern hemisphere countries (Table 1). In 2010/2011 season, the percentage of laboratory-confirmed deaths was 74.2% of that in the previous season. In Croatia, the mortality in the 2010/2011 season until March 14, 2011 was similar to that in 2009/2010 (0.71/100 000 vs 0.58/100 000; χ^2^_1_ = 0.43, *P* = 0.20). This was despite the fact that in 2009/2010 season fewer than 20 000 persons (0.4% of the population) were vaccinated, due to delayed administration of influenza vaccine H1N1A 09, compared with more than 550 000 (more than 12% of population) in the second season (Figure 1).

In 2010/2011, an even higher mortality rate than in Croatia was recorded in Greece, which also had a higher mortality in the 2009/2010 season (Tables 1 and 2). In 2010/2011, only 3.5% of Greek population was vaccinated (9). In contrast, the UK, in which more than 70% of the risk-group population was vaccinated, also had a high laboratory-confirmed mortality in 2010/2011 (22).

**Table 2:** Laboratory-confirmed deaths from influenza AH1N1 09 per months in 2010/2011 season*

		Season 2010/2011											
Country		2010								2011			total
	Population (million)	V	VI	VII	VIII	IX	X	XI	XII	I	II	III	
**Albania**	3.2									1			**1**
**Bosnia and Herzegovina**	4.0									10	6	2	**18**
**Bulgaria**	7.6								1	30	70	27	**128**
**Croatia**	4.5								1	9	15	1	**26**
**Greece**	11.2									15	110	37	**162**
**Hungary**	10.0									4	31	36	**71**
**Kosovo**	2.1										2		**2**
**Macedonia**	2.1									5	4	8	**17**
**Montenegro**	0.7									5	1	1	**7**
**Romania**	21.5								3	3	12	14	**32**
**Serbia**	7.5									5	11	8	**24**
**Slovenia**	2.0									1			**1**
**Total South-Eastern Europe**	**76.4**	**0**	**0**	**0**	**0**	**0**	**0**	**0**	**5**	**88**	**262**	**134**	**489**
									1.0	18.0	53.6	27.4	100%
**Europe without CIS countries**	**534.1**	**7**	**2**	**0**	**0**	**1**	**0**	**5**	**42**	**617**	**815**	**347**	**1836**
		0.4	0.1					0.3	2.3	33.6	44.4	18.9	100%
**Argentina**	40.5				22								**22**
**Australia**	21.7		1	2	3	13	4						**23**
**Chile**	17			5	4	6	5						**20**
**New Zealand**	4.2			2	14	4							**20**
**Paraguay**	6.8										9		**9**
**South Africa**	48												**0**
**Uruguay**	3.5												**0**
**Total Southern hemisphere** (7 countries)	**141.7**	**0**	**1**	**9**	**43**	**23**	**9**	**0**	**0**	**0**	**9**	**0**	**94**
			1.1	9.6	45.7	24.5	9.6				9.6		100%
**Equatorial area** (51 countries)**^†^**	**1776.8**	**61**	**80**	**230**	**294**	**573**	**106**	**110**	**121**	**13**	**177**	**88**	**1853**
		3.3	4.3	8.1	15.9	30.9	5.7	5.9	6.5	0.7	9.5	4.7	100%
**Northern hemisphere** (69 countries)**^†^**	**1823.4**	**34**	**56**	**27**	**0**	**1**	**5**	**51**	**118**	**801**	**1040**	**700**	**2833**
		1.2	2.0	1.0			0.2	1.8	4.2	28.3	36.7	24.7	100%
**The world** (127 countries)**^†^**^‡^	**3743.4**	**95**	**137**	**266**	**337**	**597**	**120**	**161**	**239**	**814**	**1226**	**800**	**4780**
		2.0	2.9	5.5	7.1	12.5	2.5	3.4	5.0	17.0	25.6	16.7	100%
**Total Western hemisphere** (35 countries)**^†^**	**904.0**	**64**	**78**	**87**	**9**	**26**	**6**	**95**	**24**	**47**	**136**	**184**	**756**
		8.5	10.3	11.5	1.2	3.4	0.8	12.6	3.2	6.2	18.0	24.3	100%
**Total Eastern hemisphere** (92 countries)**^†^**	**2838.0**	**31**	**59**	**179**	**328**	**571**	**114**	**66**	**215**	**767**	**1090**	**604**	**4024**
		0.8	1.5	4.4	8.2	14.2	2.8	1.6	5.3	19.1	27.1	15.0	100%
**The world** (214 countries)^§^	**6724.3**	**95**	**137**	**267**	**315**	**597**	**119**	**168**	**235**	**863**	**1351**	**798**	**4945**
		1.9	2.8	5.4	6.4	12.1	2.4	3.4	4.8	17.5	27.3	16.1	100%
**Total Northern America** (2 countries)	**340.0**	**0**	**0**	**0**	**0**	**0**	**2**	**8**	**16**	**43**	**99**	**99**	**267**
		0	0	0	0	0	0.7	3.0	6.0	16.1	37.1	37.1	100%

*Shaded fields indicate enhanced seasonal flu activity; CIS countries, Commonwealth of Independent States. Source: references 7-67.

†Countries with total mortality rates in both seasons <0.10/100 000 were excluded from calculation.

‡Reliable data on influenza-related deaths were available for 127 countries.

§Calculations for all 214 countries, regardless of the number of reported deaths.

Poland is the only European country that refused the purchase of type-specific vaccine against influenza AH1N1 2009 in the 2009/2010 season. If the last available vaccination coverage for Poland from 2008 is used to estimate the current vaccination coverage (10% of the population older than 65 years), it is clear that the type-specific vaccine cannot be the reason for significantly lower overall laboratory-confirmed influenza mortality in Poland (0.78/100 000) than in the UK (1.55/100 000) in both seasons (χ^2^_1_ = 112.2, *P* < 0.001) (38,81).

**Figure 2:**
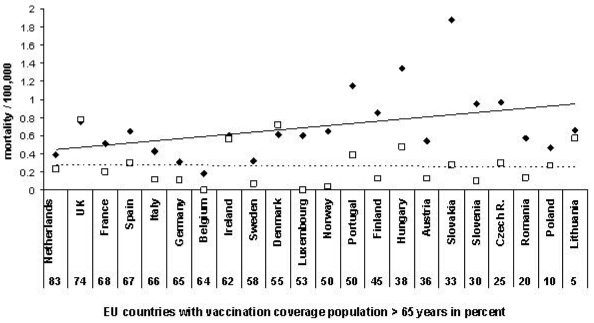
Mortality rates of laboratory confirmed deaths of influenza AH1N1 2009 in 2009/10 season (closed rhombs and full line) and 2010/11 season (open squares and broken line) in relation to estimated vaccination coverage in EU countries. Asterisks and numbers indicate the data for the UK, the country with high vaccination coverage, and Poland (low vaccination coverage), where Poland had significantly lower mortality than the UK in both seasons (*P* < 0.001) despite lower vaccination coverage. Sources: references 2,3,5-14,21-39,81.

As the current data on vaccine coverage are not available for 22 EU countries, the data for the seasonal influenza in 2008 could be used (Figure 2). Since our practice in the field shows that the desired vaccination coverage is not achieved rapidly and that the habit of vaccination is not rapidly lost, similar vaccination coverage could be expected for the 2010/11 season. In 2009/2010 season, when type-specific vaccines against influenza AH1N1 2009 were not timely available in EU countries, laboratory-confirmed deaths mortality rate in the >65 age group linearly increased as the vaccination coverage decreased (Figure 2). In 2010/2011 season, when the vaccines were available, greater vaccine coverage was not followed by a lower mortality rate (13,14,21-39,81).

The comparison of laboratory-confirmed AH1N1 2009 mortality rates among countries, even without sero-epidemiological field investigation, and detailed analysis by time, age, disability, geographic distribution, and with no valid data on the volume of applied non-pharmaceutical and pharmaceutical measures (vaccines and antivirals for preventive purposes) demonstrated that anti-epidemic protection measures by type-specific vaccines against AH1N1 2009 influenza virus in 2010/11 in SEE, Europe, and all Northern hemisphere countries did not result in a lower expected mortality rate than in Southern hemisphere countries.

**Figure 3:**
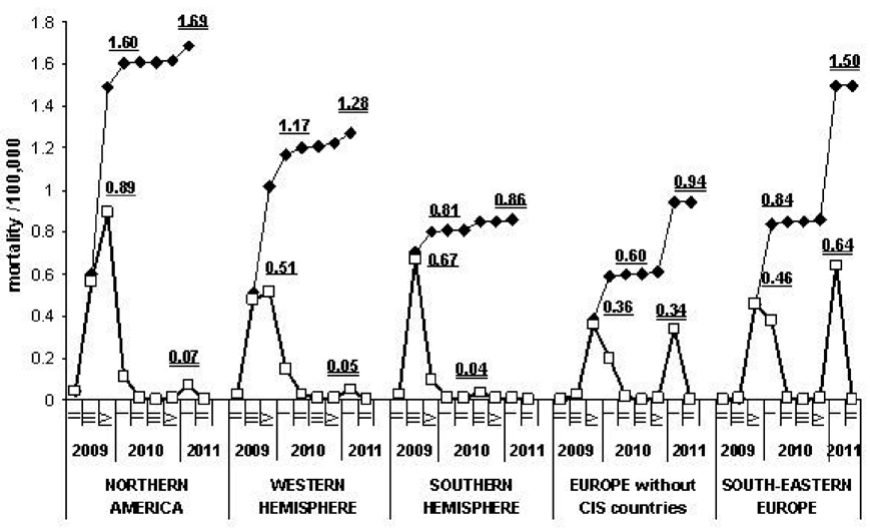
Cumulative (closed rhombs) and quarterly (open squares) mortality rates of laboratory confirmed deaths of influenza AH1N1 2009 in different global areas. Numbers present the mortality rates for 2009/10 season (single underlined) and 2010/11 (double underlined). Sources: references 7-67.

Countries of the Western hemisphere, which had a higher rate of laboratory-confirmed influenza AH1N1 2009 deaths, ie, greater active infectious immunity by natural contact with influenza virus during the 2009/10 season, and thus greater estimated consumption of epidemic potential, had a significantly smaller number of laboratory-confirmed deaths in the 2010/11 season (Figure 3) than in 2009/2010, compared with the Eastern hemisphere (Table 2, Figure 3).

With the growing evidence on the questionable efficacy of influenza vaccines (86-89) and despite the lack of reliable sero-epidemiological studies, the pattern of laboratory-confirmed AH1N1 2009 mortality indicated that Western hemisphere countries achieved a higher degree of acquired active, infectious immunity than other world regions (Table 4, Figure 3). Also, the expected epidemiological effect of vaccine immunity was not achieved. This suggests that infectious immunity has greater epidemiological significance than vaccine immunity. This is supported by the fact that people older than 55 years had lower morbidity in all parts of the world (7-67), indicating that they had been actively immunized with the infectious virus AH1N1 2009 during their life. No less important is the fact that infectious immunity provides a high degree of crossed immunity to other influenza viruses (90), while vaccine immunity lasts no longer than a year, is highly type-specific, and the possibility of crossed immunity is minimal (88,89,91). We believe that these findings clearly demonstrate that, from an epidemiological point of view, the vaccination for influenza during its outbreak should be reserved for health-risk groups. Healthy people need not get vaccinated, especially not in mass vaccination.

Data obtained by type-specific diagnostic tests in the two AH1N1 2009 influenza seasons brought into the question the previous estimates of 250 000 to 500 000 annual deaths from influenza (77-79). It was estimated that in the 2009/2010 season, the overall laboratory-confirmed influenza mortality rate in 214 countries with 6.742 million population would be 0.57/100 000, ie 38 500 deaths (Table 4). Taking into consideration the Centers for Disease Control estimate that only a third of influenza deaths are laboratory-confirmed (48), this number could reach 115 500 deaths. So far in the 2010/2011 influenza season, which is at its very end, the final estimated death toll is 25 000.

In addition to AH1N1 2009 influenza virus, other influenza viruses A and B were in circulation in different parts of the world (92). In the countries that used laboratory confirmation of influenza deaths, the total mortality of other influenza deaths did not exceed that of the AH1N1 2009 influenza (13), suggesting that the total annual number of deaths from all types influenza viruses did not exceed 50 000 in 2010/2011. From this perspective, the estimates of 250 000 to 500 000 annual deaths should be replaced by 50 000 to 150 000 annual deaths.

By studying the natural experiment of the influenza AH1N1 2009 epidemics during two seasons and by using the number of laboratory-confirmed deaths, we can provide a more accurate and reliable prognosis for the upcoming 2011/2012 season. Because of the attenuation of the epidemic potential, countries of West hemisphere will not have epidemic occurrence of influenza a H1N1 2009 above the usual average of any seasonal influenza. The countries of Eastern hemisphere will have a low epidemic occurrence only in the countries and areas where the total mortality rate was below 1.25/100 000 during the previous two seasons. The equatorial area countries will have the usual epidemic activity for another season but of lower intensity than in the previous season (Table 4, Figure 3). Influenza virus AH1N1 2009 will continue its circulation around the world as the common seasonal influenza virus did at the beginning of April 2009.

## 

**Table 3:** Comparison of AH1N1 09 mortality during influenza seasons in South-Eastern Europe and in southern hemisphere, where the AH1N1 09 flu originated in 2009*

		Season 2009/2010		Season 2010/2011		Both seasons		Ratio 2010/2011 vs 2009/2010	
Country	Population (million)	number of deaths	mortality per 100 000	number of deaths	mortality per 100 000	number of deaths	mortality per 100 000	ratio	%
Albania	3.2	12	0.38	1^†^	0.03	13	0.41	1/12	8.3
Bosnia and Herzegovina	4.0	19	0.48	18	0.45	37	0.93	18/19	94.7
Bulgaria	7.6	40	0.53	128^†^	1.68	168	2.21	128/40	320.0
Croatia	4.5	32	0.71	26	0.58	58	1.29	26/32	81.3
Greece	11.2	149	1.33	162	1.45	311	2.78	162/149	108.7
Hungary	10.0	134	1.34	71^†^	0.71	205	2.05	71/134	53.0
Kosovo	2.1	14	0.67	2^†^	0.09	16	0.76	2/14	14.3
Macedonia	2.1	26	1.24	17	0.81	43	2.05	17/26	65.4
Montenegro	0.7	8	1.14	7	1.00	15	2.14	7/8	87.5
Romania	21.5	122^†^	0.57	32^†^	0.15	154	0.72	32/122	26.2
Serbia	7.5	84^†^	1.12	24^†^	0.32	108	1.44	24/84	28.6
Slovenia	2.0	19^†^	0.95	1^†^	0.05	20	1.00	1/19	5.3
Total South-Eastern Europe	76.4	659	0.86 (0.65-1.1)	489	0.64 (0.26-0.96)^†^	1148	1.50 (1.01-1.96)^‡^	489/659	74.2^‡^
Argentina	40.5	627	1.55	22^†^	0.05	649	1.60	22/627	3.5
Australia	21.7	191	0.88	23^†^	0.11	214	0.99	23/191	12.0
Chile	17	155	0.91	20^†^	0.12	175	1.03	20/155	12.9
New Zealand	4.2	35	0.83	20	0.48	55	1.31	20/35	57.1
Paraguay	6.8	53	0.78	9^†^	0.13	62	0.91	9/53	17.0
South Africa	48	93	0.19	0	0	93	0.19	0/93	0
Uruguay	3.5	33	0.94	0	0	33	0.94	0/33	0
Total southern hemisphere	141.7	1187	0.84 (0.5-1.2)	94	0.07 (-0.02-0.28)^†^	1281	0.91 (0.6-1.4)	94/1187	7.9

*Numbers in brackets are 95% confidence intervals.

†*P* < 0.001 vs previous year (χ^2^ test).

‡*P* < 0.001 vs Southern hemisphere (χ^2^ test).

## References

[1] Kamps SB, Hoffman C, Preiser W. Influenza report 2006. Flying Publisher. Available from: www.InfluenzaReport.com*.* Accessed: April 10, 2011.

[2] WHO. Human infection with pandemic (H1N1) 2009 virus: updated interim WHO guidance on global surveillance, 10 July 2009. Available from: http://www.who.int/csr/resources/publications/swineflu/interim_guidance/en/index.html*.* Accessed: April 10, 2011.

[3] Centers for Disease Control and Prevention (CDC). Influenza diagnostic testing during the 2009-2010 flu season. Available from: http://www.cdc.gov/h1n1flu/diagnostic_testing_public_qa.htm Accessed: April 10, 2011.

[4] WHO. Assessing the severity of an influenza pandemic, 11 May 2009. Available from: http://www.who.int/csr/disease/swineflu/assess/disease_swineflu_assess_20090511/en/index.html Accessed: April 10, 2011.

[5] WHO. Pandemic influenza preparedness and response. WHO guidance document, April 2009. Available from: http://www.who.int/csr/disease/influenza/pipguidance2009/en/index.html Accessed: April 10, 2011. 23741778

[6] Schulze M, Nitsche A, Schweiger B, Biere B (2010). Diagnostic approach for the differentiation of the pandemic influenza A(H1N1)v virus from recent human influenza viruses by real-time PCR.. PLoS ONE.

[7] Institute for Public Health FB&H. Available from. : http://www.zzjzfbih.ba/tag/gripa/*.* Accessed: April 10, 2011.

[8] Croatian Institute of Public Health. Pandemic influenza caused by new virus A/H1N1. Available from: http://www.hzjz.hr/epidemiologija/svinjska_gripa.htm Accessed: April 10, 2011.

[9] Hellenic Center for Disease Control and Prevention, Department of Epidemiological Surveillance. Athens: KEELPNO. Weekly epidemiological report. Available from: http://www.keelpno.gr Accessed: April 10, 2011.

[10] Institute for Public Health of the Republic of Macedonia. Available from: http://www.iph.mk/index.php?lang=en*.* Accessed: April 10, 2011.

[11] Crna Gora Instiute of Public Health. Available from: http://www.ijzcg.me/*.* Accessed: April 10, 2011.

[12] Serbian Instiute of Public Health. “Dr Milan Jovanović Batut.” Pandemic influenza A (H1N1). Available from: http://www.batut.org.rs/ah1n1.html Accessed: April 10, 2011.

[13] European Centre for Disease Prevention and Control (ECDC). Available from: http://www.ecdc.europa.eu/en/Pages/home.aspx Accessed: April 10, 2011.

[14] WHO Regional Office for Europe. Influenza, Pandemic (H1N1) 2009 in the European Region. Available from: http://www.euro.who.int/en/what-we-do/health-topics/communicable-diseases/influenza/pandemic-h1n1-2009*.* Accessed: April 10, 2011.

[15] Argentina R. Ministerio de Salud da la Nacion, Vigilancia de infecciones respiratorias agudas en Argentina. Available from: http://www.msal.gov.ar*.* Accessed: April 10, 2011.

[16] Australian Government, Department of Health and Ageing, Update bulletins for Pandemic (H1N1) 2009. Available from: http://www.health.gov.au/internet/main/publishing.nsf/Content/cda-surveil-ozflu-flucurr.htm Accessed: April 10, 2011.

[17] Ministerio de Salud de Chile. Informe de influenza. Available from: http://www.pandemia.cl/ Accessed: April 10, 2011.

[18] New Zealand Ministry of Health. Pandemic Influenza H1N1 2009 (swine flu). Available from: http://www.moh.govt.nz/moh.nsf/indexmh/influenza-a-h1n1-news-media#mediareleases*.* Accessed: April 10, 2011.

[19] National Institute for Communicable Diseases. A Division of the National Health Laboratory Service, South Africa, Seasonal Influenza 2010. Available from: http://www.nicd.ac.za/ Accessed: April 10, 2011.

[20] Pan American Health Organization. Pandemic (H1N1) 2009 – Americas, regional updates, influenza. Available from: http://new.paho.org/hq/index.php?option=com_content&task=view&id=1239&Itemid=1091 Accessed: April 10, 2011.

[21] Nederlands Institut voor onderzoek van de gezondheidszorg. Available from: http://www.nivel.nl/griep/*.* Accessed: April 10, 2011.

[22] Health Protection Agency. Available from: http://www.hpa.org.uk/*.* Accessed: April 10, 2011.

[23] Gobierno de Espana. Ministerio de sanidad y politica Social, Información sobre la gripe A (H1N1). Available from: http://www.msc.es/servCiudadanos/alertas/gripeAH1N1.htm Accessed: April 10, 2011.

[24] Institut de veille sanitaire. Grippe, situation épidémiologique. Available from: http://www.invs.sante.fr/surveillance/grippe_dossier/default.htm Accessed: April 10, 2011.

[25] Ministero della Salute Italiana. Nuova influenza, Aggiornamento epidemiologico settimanale. Available from: http://www.nuovainfluenza.salute.gov.it/nuovainfluenza/nuovaInfluenza.jsp Accessed: April 10, 2011.

[26] Robert Koch Institut. Pandemische influenza (H1N1) 2009. Available from: http://www.rki.de/influenza Accessed: April 10, 2011.

[27] Centers for Disease Control and Prevention (CDC). Influenza laboratory-2010. Influenza surveillance program in Belgium. Available from: http://influenza.wiv-isp.be/Pages/Influenza.aspx Accessed: April 10, 2011.

[28] Health Protection Surveillance Centre. Ireland. Available from*:* http://www.hpsc.ie/hpsc/*.* Accessed: April 10, 2011.

[29] Swedish Institute for Infectious Diseases Control. Available from: http://www.smittskyddsinstitutet.se/in-english/*.* Accessed: April 10, 2011.

[30] State Serum Institute. Denmark. Available from: http://www.ssi.dk/Aktuelt/Nyhedsbreve/INFLUENZA-NYT/2010-2011.aspx Accessed: April 10, 2011.

[31] Norwegian Institute of Public Health. Available from: http://www.fhi.no/eway/?pid=238 Accessed: April 10, 2011.

[32] Portugal Institute of Public Health. Available from: http://www.insa.pt/sites/INSA/Portugues/AreasCientificas/Epidemiologia/Paginas/ActividadeGripal.aspx Accessed: April 10, 2011.

[33] National Institute for Health and Welfare. Finland. Available from: http://www.ktl.fi/ttr/gen/rpt/h1n1.pdf Accessed: April 10, 2011.

[34] Hungarian Ministry of Health. Available from: http://www.eum.hu/influenza Accessed: April 10, 2011.

[35] Österreiche Bundesministerium für Gesundheit. Available from: http://www.bmgfj.gv.at/*.* Accessed: April 10, 2011.

[36] Public Health Authority of the Slovak Republic. Available from: http://www.uvzsr.sk/index.php?option=com_content&view=category&layout=blog&id=58&Itemid=64 Accessed: April 10, 2011.

[37] Czech Republic. Státní zdravotní ústav, Aktuality. Available from: http://www.szu.cz/aktuality?lchan=1&lred=1 Accessed: April 10, 2011.

[38] National Institute of Public Health. Influenza and influenza-like illness in Poland. Available from: http://www.pzh.gov.pl/oldpage/epimeld/grypa/aindex.htm Accessed: April 10, 2011.

[39] Centre for Communicable Diseases and AIDS. Lithuania. Available from: http://www.ulac.lt/index.php?pl=107&ppl=8#2010_2011 Accessed: April 10, 2011.

[40] Federal Office of Public Health (Switzerland). Available from: http://www.bag.admin.ch/influenza/01120/index.html?lang=en Accessed: April 10, 2011.

[41] Health Board. Estonia. Available from: http://www.terviseamet.ee/info/uudised/u/artikkel/5-nadal-ulemiste-hingamisteede-viirusnakkustesse-haigestumine-hakkab-stabiliseeruma.html*.* Accessed: April 10, 2011.

[42] Directorate of Health. Iceland. Available from: http://www.influensa.is/Pages/843 Accessed: April 10, 2011.

[43] State Agency. “Infectology Centre of Latvia,” Latvia. Available from: http://www.lic.gov.lv/index.php?p=1327&pp=10799&lang=258*.* Accessed: April 10, 2011.

[44] Russian Federation, Federal Surveillance Service for Communicable Diseases. Information about morbidity caused by highly pathogenic influenza viruses. Available from: http://www.rospotrebnadzor.ru/press_center/press/3121/*.* Accessed: April 10, 2011.

[45] Ministry of Health of Ukraine. Available from: http://www.moz.gov.ua/ua/portal/allnews/*.* Accessed: April 10, 2011.

[46] Israel Center for Disease Control. Influenza Activity, weekly reports 2009-2010. Available from: http://www.health.gov.il/english/pages_E/default.asp?maincat=15*.* Accessed: April 10, 2011.

[47] Turkey, T.C. Saglik Bakanligli. Available from: http://www.grip.gov.tr/*.* Accessed: April 10, 2011.

[48] Centers for Disease Control and Prevention. USA. A weekly influenza surveillance report prepared by the influenza division. Available from: http://www.cdc.gov/flu/weekly/index.htm Accessed: April 10, 2011.

[49] Estados Unidos Mexicanos, Estadísticas de la Epidemia Influenza A (H1N1). Available from: http://portal.salud.gob.mx/contenidos/noticias/influenza/estadisticas.html*.* Accessed: April 10, 2011.

[50] Public Health Agency of Canada. H1N1 flu virus, weekly FluWatch reports. Available from: http://www.phac-aspc.gc.ca/fluwatch/09-10/index-eng.php Accessed: April 10, 2011.

[51] WHO. Situation updates – Pandemic (H1N1) 2009. Available from: http://www.who.int/csr/disease/swineflu/updates/en/index.html*.* Accessed: April 10, 2011.

[52] WHO. Regional Office for South-East Asia, Pandemic H1N1 2009. Available from: http://www.searo.who.int/EN/Section10/Section2562.htm Accessed: April 10, 2011.

[53] WHO Western Pacific Region, Influenza A (H1N1) 2009. Available from: http://www.wpro.who.int/health_topics/h1n1/*.* Accessed: April 10, 2011.

[54] WHO Regional Office for the Eastern Mediterranean. Pandemic (H1N1) 2009. Available from: http://www.emro.who.int/csr/h1n1/*.* Accessed: April 10, 2011.

[55] WHO Regional Office for Africa. Pandemic Influenza (H1N1) 2009 – Current situation in the WHO African Region. Available from: http://www.afro.who.int/en/clusters-a-programmes/dpc/epidemic-a-pandemic-alert-and-response/programme-components/epidemic-readiness-and-intervention/pandemic-influenza-h1n1-2009-current-situation-in-the-who-african-region.html*.* Accessed: April 10, 2011.

[56] Ministerio da Saude Brasil, Secretaria de Vigilancia em Saude, Influenza Pandęmica (H1N1) 2009 – monitoramento da síndrome respiratória aguda grave (SRAG) em hospitalizados. Available from: http://portal.saude.gov.br/portal/arquivos/pdf/informe_influenza_8_agosto19_8_10.pdf*.* Accessed: April 10, 2011.

[57] Department of Health The government of the Hong Kong Special Administrative Region. Available from: http://www.chp.gov.hk/en/guideline1_year/29/134/441/518.html*.* Accessed: April 10, 2011.

[58] La Dirección General de Epidemiología (DGE) del Ministerio de Salud Peru. Available from: http://www.dge.gob.pe/influenza/AH1N1/*.* Accessed: April 10, 2011.

[59] Instituto Pedro Kouri Direccion nacional de epidemiologia Ministerio de salud publica La Habana, Cuba. Available from: http://files.sld.cu/ipk/files/2010/03/bol02-10.pdf Accessed: April 10, 2011.

[60] Chinese National Influenza Center. Influenza weekly report, overview. Available from: http://www.cnic.org.cn/eng/ Accessed: April 10, 2011.

[61] Ministry of Health. Labour and Welfare, Influenza A (H1N1) – current situation in Japan. Available from: http://www-bm.mhlw.go.jp/english/topics/influenza_a/index.html*.* Accessed: April 10, 2011.

[62] Korea Centers for Disease Control and Prevention. Available from: http://www.cdc.go.kr/eng/english/kcdc_01.htm Accessed: April 10, 2011.

[63] Ministry of Health Singapore. Current influenza situation in Singapore. Available from: http://www.moh.gov.sg/mohcorp/diseases.aspx?id=24616 Accessed: April 10, 2011.

[64] Centers for Disease Control (Taiwan). Available from: http://www.cdc.gov.tw/*.* Accessed: April 10, 2011.

[65] Government of India. Press Information Bureau. Available from: http://pib.nic.in/h1n1/h1n1.asp*.* Accessed: April 10, 2011.

[66] Kementerian Kesihatan Malaysia. Influenza A (H1N1). Available from: http://h1n1.moh.gov.my/ Accessed: April 10, 2011.

[67] Pacific Public Health Surveillance Network. Available from: http://www.spc.int/phs/PPHSN/Surveillance/Syndromic.htm*.* Accessed: April 10, 2011.

[68] Population Division of the Department of Economic and Social Affairs of the United Nations Secretariat. World Population Prospects: the 2008 revision. Available from: http://esa.un.org/unpp*.* Accessed: April 10, 2011.

[69] MMWR swine Influenza A (H1N1) infection in two children – Southern California, March-April 2009. Available from: http://www.cdc.gov/mmwr/preview/mmwrhtml/mm5815a5.htm Accessed: April 10, 2011. 19390508

[70] WHO. Swine influenza, statement by WHO Director-General, Dr Margaret Chan. Available from: http://www.who.int/mediacentre/news/statements/2009/h1n1_20090425/en/index.html Accessed: April 10, 2011.

[71] Butler D (2009). Swine flu goes global.. Nature.

[72] Fraser C, Donnelly CA, Cauchemez S, Hanage WP, Van Kerkhove MD, Hollingsworth TD (2009). Pandemic potential of a strain of influenza A (H1N1): early findings.. Science.

[73] Dunn FL (1958). Pandemic influenza in 1957; review of international spread of new Asian strain.. JAMA.

[74] Barry JM (2009). Observations on Past Influenza Pandemics.. Disaster Med Public Health Prep.

[75] Hine DD. The 2009 Influenza Pandemic, An independent review of the UK response to the 2009 influenza pandemic. London: Cabinet Office; 2010.

[76] Bowcott O. Swine flu could kill 65,000 in UK, warns chief medical officer. Available from: http://www.guardian.co.uk/world/2009/jul/16/swine-flu-pandemic-warning-helpline*.* Accessed: April 10, 2011.

[77] World Health Organization. Assessing the severity of an influenza pandemic. Available from: http://www.who.int/csr/disease/swineflu/assess/disease_swineflu_assess_20090511/en/index.html*.* Accessed: April 10, 2011.

[78] CDC estimates of 2009 H1N1 influenza cases. Hospitalizations and deaths in the United States. Available from: http://www.cdc.gov/h1n1flu/estimates_2009_h1n1.htm*.* Accessed: April 10, 2011.

[79] Harper AS, Fukuda K, Uyeki TM, Cox NJ, Bridges CB. Prevention and control of influenza. Available from: http://www.cdc.gov/mmwr/preview/mmwrhtml/rr5408a1.htm*.* Accessed: April 10, 2011.

[80] Smoljanović M. Pandemija gripe A H1N1 09. Let the facts speak [in Croatian]. Available from: http://www.plivamed.net/aktualno/clanak/3261/Pandemija-gripe-A-H1N1-09-Neka-govorecinjenice.html Accessed: April 10, 2011.

[81] WHO. Weekly epidemiological record. Available from: http://www.who.int/wer/2009/wer8449.pdf Accessed: April 10, 2011.

[82] WHO. Recommended viruses for influenza vaccines for use in the 2010-2011 northern hemisphere influenza season, 2010. Available from: http://www.who.int/csr/disease/influenza/recommendations2010_11north/en/index.html*.* Accessed: April 10, 2011. 20210260

[83] New Zealand Ministry of Health. Pandemic influenza immunisation, 2010. Available from: http://www.moh.govt.nz/moh.nsf/pagesmh/10268/$File/pandemic-influenza-immunisation-cabinet-paper-oct09.pdf*.* Accessed: April 10, 2011.

[84] NZPA. Fluvaccine given to more than 1M people this year, 15.Jun 2010. Available from: http://www.3news.co.nz/Flu-vaccine-given-to-more-than-1M-people-this-year/tabid/423/articleID/161050/Default.aspx?ArticleID=161050&utm_source*.* Accessed: April 10, 2011.

[85] Argentina MOH official reports for H1N1. Manzur presented the results of the campaign against influenza A [in Spanish]. Available from: http://www.infobae.com/notas/533538-Manzur-presento-los-resultados-de-la-campana-contra-la-gripe-A.html Accessed: April 10, 2011.

[86] Mereckiene J, Cotter S, Nicoll A, Levy-Bruhl D, Ferro A, Tridente G (2008). National seasonal influenza vaccination survey in Europe, 2008. Euro Surveill.

[87] Vardavas R, Breban R, Blower S (2010). A universal long-term flu vaccine may not prevent severe epidemics.. BMC Res Notes..

[88] Kissling E, Valenciano M, I-MOVE case-control studies team (2011). Early estimates of seasonal influenza vaccine effectiveness in Europe, 2010/11: I-MOVE, a multicentre case-control study. Euro Surveill.

[89] Savulescu C, Jimenez-Jorge S, de Mateo S, Ledesma J, Pozo F, Casas I (2011). Effectiveness of the 2010-2011 seasonal trivalent influenza vaccine in Spain: preliminary results of a case-control study. Euro Surveill.

[90] Wrammert J, Koutsonanos D, Li GM, Edupuganti S, Sui J, Morrissey M (2011). Broadly cross-reactive antibodies dominate the human B cell response against 2009 pandemic H1N1 influenza virus infection.. J Exp Med.

[91] Puig-Barbera J. 2010-2011 influenza seasonal vaccine, preliminary mid-season effectiveness estimates: reason for concern, confounding, or are we following the right track?(Editorial)Euro Surveill 2011 16 pii:19821 21435331 10.2807/ese.16.11.19821-en

[92] WHO. Global influenza virological surveillance. Available from: http://www.who.int/csr/disease/influenza/influenzanetwork/en/index.html Accessed: April 10, 2011.

